# A Retrospective Study of the Prevalence of Maxillary Sinus Cysts Incidentally Detected on MRI Among Non-Symptomatic Caucasian Population

**DOI:** 10.3390/jcm14196756

**Published:** 2025-09-24

**Authors:** Piotr Rot, Sandra Krzywdzińska, Paweł Rozbicki, Marta Aleksandra Kwiatkowska, Marta Kania-Pudło, Arkadiusz Zegadło, Dariusz Jurkiewicz, Karolina Dżaman, Maria Sobol

**Affiliations:** 1 Clinic of Otolaryngology and Oncological Otolaryngology with Clinical Department of Cranio-Maxillofacial Surgery, Military Institute of Medicine—National Research Institute, 04-141 Warsaw, Poland; skrzywdzinska@wim.mil.pl (S.K.); prozbicki@wim.mil.pl (P.R.); mkwiatkowska1@wim.mil.pl (M.A.K.); djurkiewicz@wim.mil.pl (D.J.); 2 Clinical Department of Radiology, Military Institute of Medicine—National Research Institute, 04-141 Warsaw, Poland; mkania-pudlo@wim.mil.pl (M.K.-P.); azegadlo@wim.mil.pl (A.Z.); 3 Department of Otolaryngology, The Medical Centre of Postgraduate Education, 01-813 Warsaw, Poland; kfrydel@poczta.onet.pl; 4 Department of Biophysics, Physiology and Pathophysiology, Medical University of Warsaw, 02-901 Warsaw, Poland

**Keywords:** incidental findings, maxillary sinus, MRI, mucosal thickening, retention cysts, sinus abnormalities

## Abstract

Maxillary sinus abnormalities, including retention cysts and mucosal thickening, are often incidental findings and may be present in up to 35.6%, depending on imaging modality and population characteristics. To date, few studies have explored the appearance of maxillary sinus retention cysts using MRI. **Aim:** This study provides the first large-scale MRI-based assessment of these lesions, with the aim of evaluating the prevalence and characteristics of maxillary sinus abnormalities detected incidentally on head MRI scans, particularly focusing on retention cysts and mucosal changes. **Materials and Methods:** A retrospective analysis of 3092 head MRI scans obtained between 2023 and 2024 was conducted to assess the prevalence and characteristics of maxillary sinus abnormalities. The mean patient age was 54.5 ± 18.6 years (median 56; range 18–99 years), with 1,825 women (59%). Statistical power exceeded 83% to detect differences of at least 10% in the prevalence of cysts between age groups (α = 0.05). A simplified MRI-based sinus assessment scale was used to categorize findings. **Results:** Out of 3092 scans, 1995 (64.5%) showed normal sinuses, 817 (26.4%) had mucosal thickening < 5 mm, 116 (3.8%) presented with retention cysts without other pathology, 100 (3.2%) exhibited moderate changes, and 64 (2.1%) had severe changes. Cysts were significantly more frequent in men (7.5%) than in women (4.4%) (*p* < 0.001). Additionally, maxillary sinus involvement differed significantly between sexes, with a higher prevalence in men. **Conclusions:** Incidental maxillary sinus findings are common and often benign. A comprehensive diagnostic approach is essential, especially for unilateral lesions near tooth roots. Conservative management remains appropriate for asymptomatic patients, but ongoing monitoring and patient education are key to preventing complications.

## 1. Introduction

Maxillary sinus retention cysts (MSRCs) are among the most common lesions identified during imaging of the paranasal sinuses and are frequently found incidentally in asymptomatic individuals [1,2,3]. However, differentiating MSRCs from jaw cysts can be challenging due to overlapping anatomical locations and similar radiographic appearances. In such cases, clinical symptoms may offer limited diagnostic value, highlighting the essential role of radiological evaluation in achieving an accurate differential diagnosis [4]. Moreover, the distinction between MSRCs and maxillary sinus pseudocysts can also be problematic. MSRCs arise from obstruction of the excretory ducts of the seromucous glands within the sinus mucosa. Histologically, these true cysts are characterized by a thin epithelial lining. In contrast, pseudocysts lack an epithelial wall and instead result from diffuse subepithelial accumulation of inflammatory exudate within the sinus mucosa. These cysts are benign, dome-shaped, or rounded structures most commonly located on the floor of the maxillary sinus [3,5,6,7,8]. On radiographs, they typically present as uniform, dense, cup-shaped, or “rising sun” opacities with sharply defined borders that follow the contours of the surrounding bone [6,9,10]. Their development is widely attributed to the obstruction of the ducts of seromucous glands, resulting in the accumulation of mucous or serous fluid within the sinus mucosa and the subsequent formation of epithelial-lined cysts [5,6,7].

The prevalence of MSRCs in the general population varies widely, ranging from 3.2% to 35.6%, depending on imaging modality and population characteristics [1,11]. Although these lesions are most often considered incidental and clinically insignificant, recent studies have challenged this view by demonstrating associations between MSRCs and various sinonasal symptoms, including facial pain or pressure, headache, nasal discharge, and postnasal drip [1,12,13,14]. These findings suggest that MSRCs may not always be harmless and may contribute to or exacerbate sinonasal complaints in certain patients.

Despite their high prevalence, the natural history of MSRCs remains somewhat unpredictable. While the majority are self-limiting, with spontaneous regression or complete resolution observed in approximately 30% of cases [13], a small proportion may gradually increase in size over time [6]. About 60% remain unchanged over time. According to Moon et al. [11], patients whose cysts measure over 20 mm or present bilaterally at diagnosis are more likely to experience progression. MSRCs may enlarge enough to block the natural ostia of the maxillary sinus and, in some cases, protrude into the middle meatus by dilating these openings [14]. To date, no cases have been documented in which they erode bony walls or extend into the inferior meatus. The size of cysts alone does not appear to correlate consistently with symptom severity, and several studies have failed to establish a statistically significant relationship between cyst dimensions and clinical presentation [6,7,9]. In addition to symptomatic presentation, anatomical and inflammatory factors have been implicated in the pathogenesis of MSRCs. Studies have indicated that osteomeatal complex obstruction (OMCO), abnormalities of the middle turbinate (AMT), and nasal septal deviation (NSD) may contribute to impaired sinus drainage and persistent mucosal inflammation, thereby facilitating cyst formation [1,15,16]. Patients with MSRCs have also been shown to have higher Lund-Mackay scores compared to individuals without cysts, suggesting a possible link between the presence of cysts and underlying chronic rhinosinusitis [2,16]. Although barotrauma, dental pathology, allergic rhinitis, and prior sinus infections have all been proposed as contributing etiological factors, the precise mechanisms behind MSRC development remain incompletely understood [12,16].

Management of MSRCs remains a subject of clinical debate. No intervention is usually necessary, as the lesions often resolve spontaneously over time [17]. Asymptomatic lesions are typically monitored without intervention, but surgical treatment is commonly recommended when symptoms are clearly attributable to the cyst [12,14,18]. Historically, cyst management involved procedures such as puncture and aspiration through the inferior meatus or surgical excision via the Caldwell-Luc approach [18,19]. However, these methods have largely been replaced by less invasive endoscopic techniques. Middle meatal antrostomy (MMA), often combined with manipulation of the ostiomeatal complex (OMC), has become the preferred surgical strategy [12,14,20]. Nevertheless, MMA may offer limited access to certain areas of the maxillary sinus, thus leading to an incomplete removal of lesions or their recurrence [3,21].

Paranasal sinus computed tomography (PNsCT) is typically used to evaluate sinus lesions and associated anatomical variations, but with the growing use of magnetic resonance imaging (MRI) for neurologic and maxillofacial conditions, incidental detection of MSRCs has become increasingly common in MRI as well [1,22,23]. While MRI provides excellent soft tissue contrast and avoids radiation exposure, its role in the evaluation of paranasal sinus abnormalities remains less well-defined than that of CT [24]. Nonetheless, MSRCs are often identified during MRI scans performed for unrelated reasons. In one of the studies, such cysts were identified in 5.8% of orthodontic patients through panoramic radiography [17]. Their presence can create challenges for certain surgical techniques, particularly subcutaneous facial emphysema performed through a lateral window or with transcrestal osteotomes [25,26].

The clinical significance of MSRCs detected incidentally on MRI remains uncertain, especially in the absence of sinonasal complaints [1,27,28]. Some authors argue that these findings represent normal anatomical variations or innocuous mucosal changes, while others suggest that they may reflect subclinical or early-stage sinonasal disease [1,12]. Furthermore, there is no unified consensus on whether incidentally discovered MSRCs warrant further investigation, monitoring, or intervention, especially in asymptomatic patients. Radiological evaluations alone often cannot determine the clinical impact of a cyst, and anatomical variations may play a key role in whether a cyst becomes symptomatic.

In this context, a better understanding of the prevalence, characteristics, and clinical relevance of MSRCs discovered incidentally on MRI is essential.

A critical appraisal of the current literature on incidental findings detected by magnetic resonance imaging (MRI) indicates that paranasal sinus abnormalities are rarely subjected to systematic evaluation or graded according to severity. Existing studies are frequently based on heterogeneous cohorts, and in some cases, sinus alterations are omitted altogether, as the primary focus lies outside the scope of sinonasal pathology. The present investigation constitutes the first large-scale MRI-based analysis specifically targeting these lesions, aiming to determine the prevalence and morphological characteristics of maxillary sinus abnormalities incidentally identified on head MRI, with particular emphasis on retention cysts and mucosal changes [29,30].

## 2. Materials and Methods

A retrospective analysis was conducted on the results of 3092 magnetic resonance imaging (MRI) scans of the head performed on patients with various neurological indications unrelated to symptoms of the respiratory system or paranasal sinuses. Head MRIs performed between 2023 and 2024 at WIM-PIB were included in the analysis. The study was conducted in accordance with the STROBE guidelines. All scans were analyzed anonymously. Head MRIs were performed at the Military Medical Institute using the following devices:

- 3.0T Discovery MR750, GE, serial number UA0190, installed in the Magnetic Resonance Laboratory of the Department of Medical Radiology at the Military Medical Institute-National Research Institute in 2013.
- 1.5T Signa Artist, GE, serial number PM067, installed in the Magnetic Resonance Laboratory at the Department of Medical Radiology at the Military Medical Institute-National Research Institute in 2022.

These examinations were performed using standard head MR examination protocols in FSE T1-, T2-dependent, FLAIR, SWAN, and DWI/ADC sequences in transverse projections and T2-weighted sequences in frontal and sagittal projections. Some of the examinations were additionally performed with the administration of an intravenous contrast agent in 3DT1 gradient sequences and FSE T1-dependent sequences in transverse projections. Cysts in the paranasal sinuses are best visualized on FSE T2-weighted images. Additional assessment includes contrast enhancement, fluid levels in the sinuses, and mucosal thickening. The choice between a 1.5T and a 3.0T scanner does not affect the assessment of the paranasal sinuses; both scanners provide similar visualization of paranasal sinus diseases. Among the 1002 examinations performed on the 1.5 T system, pathological changes were detected in 351 cases. Among the 2090 examinations performed on the 3.5 T system, pathological changes were detected in 746 cases. The difference in pathology detection rates between the two systems was not statistically significant (*p* = 0.718).

The inclusion criteria for the study were age ≥ 18 years, the presence of a complete set of MR images of the head, including the maxillary sinuses, and no history of diagnosed inflammatory sinus disease. The exclusion criteria were artifacts preventing unambiguous assessment of the maxillary sinuses, status after sinus surgery, and the presence of systemic diseases affecting the sinus image (e.g., asthma, cystic fibrosis, granulomatosis with polyangiitis).

A modified scale for assessing maxillary sinus inflammation severity based on the widely adopted and validated Lund–Mackay scale (LM scale) for CT was used for evaluating chronic changes in the paranasal sinuses. The MRI scale represents a hybrid approach, combining the structure of the Lund–Mackay (LM) system (widely used in CT-based staging) with the quantitative mucosal thickness criteria proposed by Newman et al. [31] In the Newman scale, mucosal thickening in the paranasal sinuses is scored as 0 = none to 1 mm, 1 = 2–5 mm, 2 = 6–9 mm, and 3 = >9 mm. In our modification we retained Newman’s gradation for mild, moderate, and severe inflammatory changes (MRI grades 1–3), which improves reproducibility on MRI where mucosal thickness can be directly measured; maintained the LM principle of scoring based on the extent of sinus involvement; added MRI grade 4 for nonspecific/ambiguous lesions (to avoid forced misclassification); and introduced MRI grade 5 for maxillary sinus cysts, which MRI can differentiate with high confidence [31].

### 2.1. Statistical Analysis

All calculations presented in the study were performed using Statistica 13.0 software (Dell Software Inc., Round Rock, TX, USA) and Microsoft^®^ Excel 16.89.1 software (Microsoft Corporation, Redmond, WA, USA). The quantitative variables were summarized using descriptive statistics: the mean, the standard deviation, the median, and the range. The distribution of each variable was tested for consistency with the normal distribution using the Shapiro–Wilk test. The nonparametric Mann–Whitney test was performed as the variables were not normally distributed. The categorical data were presented as frequencies and percentages. The chi-square test was used to compare the significance of categorical independent variables (such as sex and age range). Univariate logistic regression was used to evaluate all factors potentially associated with MSRCs prevalence. Significant factors in univariate analysis were included in multivariate backward stepwise logistic regression analysis. The results were presented as odds ratios (ORs) with 95% confidence intervals (Cls). A *p*-value less than 0.05 was considered statistically significant, as all predictors were tested simultaneously within a single model. Univariate analyses were adjusted for multiple comparisons within each family of related hypotheses using the Bonferroni correction. Since different subgroups were analyzed, these were treated as independent scientific questions. Specifically, sex-related multiple comparisons were performed within the group of patients with alterations in other (non-maxillary) paranasal sinuses, and adjustments were made accordingly (α = 0.025). There were no missing data in the study. For a group of 3092 patients, we received at least 83% power to detect differences of at least 10% in the prevalence of cysts between age groups, with a significance level set at α = 0.05.

### 2.2. Ethical Approval and Consent

The study was reviewed and granted an exemption from approval by the institutional ethics committee “The Military Institute of Medicine–National Research Institute Bioethics Committee” (No. KB/24/25) due to its retrospective nature. The informed consent was waived by the reviewing ethics committee due to the retrospective nature of the study and anonymization of the clinical source data. All methods were performed in accordance with relevant guidelines and regulations. The study has been conducted in accordance with the Declaration of Helsinki.

## 3. Results

A retrospective analysis was performed on 3092 MRI scans acquired between 2023 and 2024. The mean patient age was 54.5 ± 18.6 years (median 56 years, range 18–99 years). Of the 1825 patients, 59% were women.

Using the simplified MRI sinus-assessment scale, 1995 examinations (64.5%) were classified as having normal sinuses, 817 (26.4%) showed predominantly mucosal thickening < 5 mm, 116 (3.8%) were identified as cysts with no accompanying other sinus pathology, 100 (3.2%) as showing moderate changes, and 64 (2.1%) as severe changes.

Moreover, statistically significant differences were observed in the frequency of cysts (*p* < 0.001). Cysts were diagnosed more frequently in men, 95 cases (7.5%), compared to women, 81 cases (4.4%). In addition, statistically significant differences in maxillary sinus involvement were noted between sexes, with a higher frequency observed in men (Table 1).

Moreover, patients were divided into age groups according to WHO recommendations. In this classification, mucosal thickening affecting only the maxillary sinus was most frequently observed in the 31–60-year age group, with 241 patients (49.2%) affected (Table 2).

### 3.1. MSRCs Group

MSRCs were detected in 176 patients (5.7%, Group 1). Unilateral cysts were present in 144 patients (81.8%), and bilateral cysts were present in 32 (18.2%). Among women, 71 cases (87.7%) were unilateral and 10 (12.3%) bilateral. Among men, 73 cases (76.8%) were unilateral and 22 (23.2%) were bilateral. The distribution of unilateral versus bilateral cysts did not differ significantly between sexes (*p* = 0.064).

In 63 patients (35.8%), the cysts were accompanied by changes in other paranasal sinuses. According to the simplified scale used in MRI evaluations, statistically significant differences were observed between patients with isolated cysts and those with cysts accompanied by additional sinus changes (*p* < 0.001). In the latter group, the most common findings were benign changes, predominantly mucosal thickening of less than 5 mm (Table 3).

No statistically significant differences were observed between sexes and in the distribution of findings based on the simplified MRI evaluation scale (*p* = 0.502, Table 4).

### 3.2. Findings in Patients with Alterations in Other (Non-Maxillary) Paranasal Sinuses

In the subgroup that exhibited changes limited to paranasal sinuses other than the maxillary sinus, 1995 examinations (68.4%) were classified as normal, 765 (26.2%) showed predominantly mucosal thickening < 5 mm, and cysts were the least common finding, recorded in only three patients (0.1%) (Table 5).

Based on the simplified scale used in MRI evaluations, statistically significant differences were observed when dividing the group according to sex (*p* < 0.001). Among both women and men, the most common findings were normal sinuses, observed in 72.9% of women and 61.8% of men, respectively (Table 6).

Moreover, a statistically significant difference in maxillary sinus involvement was observed when the group was divided by sex, with a higher frequency noted in men (Table 7).

### 3.3. Group with Swelling Limited to the Maxillary Sinus

In the subgroup with swelling limited to the maxillary sinus, 109 patients (22.2%) were identified with cysts only, whereas 381 patients (77.8%) exhibited other pathological changes. Among these, the most frequently observed finding was mucosal thickening of less than 5 mm, present in 332 patients (67.8%) (Table 8).

No statistically significant differences were found in the distribution of cysts by sex (*p* = 0.554, Table 9).

However, statistically significant differences were observed across age groups (*p* = 0.028). Cysts were most frequently identified in patients aged 31–60 years, representing 27% (*n* = 65) of individuals in that age group. In contrast, the lowest prevalence was observed among patients aged ≥ 90 years, with 9.4% (*n* = 5) affected in that cohort (Table 10).

### 3.4. Logistic Regression Analysis

**Figure 1:**
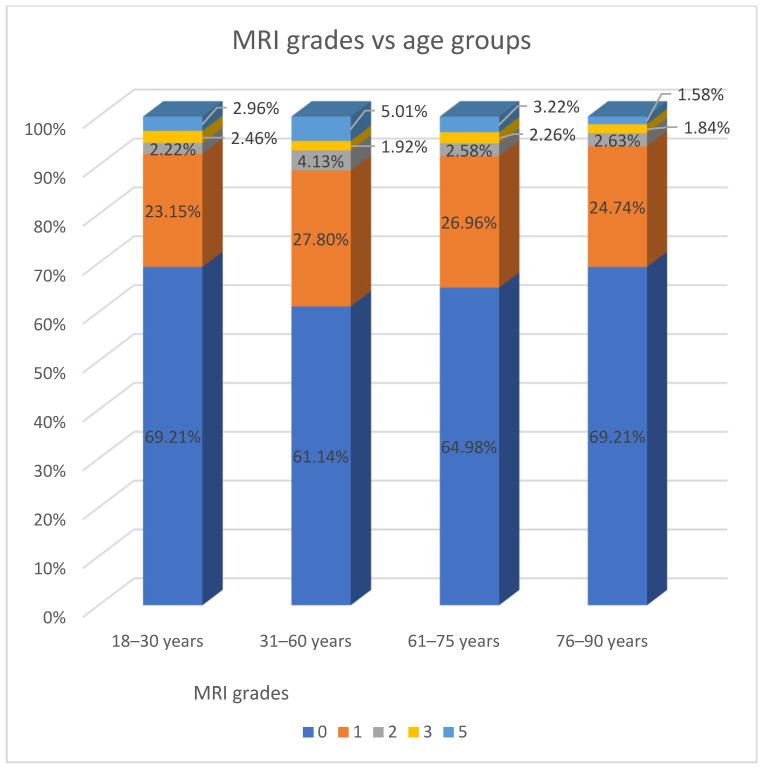
Summary of MRI grades by age group.

In the univariate logistic regression analysis, the presence of MSRCs was significantly associated with sex (*p* < 0.001) and age (*p* = 0.014). Therefore, these two variables were included in the multivariate backward stepwise logistic regression analysis. In the multivariate model, factors significantly associated with the presence of MSRCs included male sex (OR = 1.72) and older age groups compared to the 31–60 years reference group: age 61–75 years (OR = 0.70) and age 76–90 years (OR = 0.33) (Table 11 and Figure 1).

## 4. Discussion

Maxillary sinus cysts, particularly mucous retention cysts, are frequently encountered as incidental findings in radiological evaluations, most commonly in CT scans and panoramic radiography (pantomography), especially among patients undergoing dental assessments or procedures. Compared with computed tomography (CT), magnetic resonance imaging (MRI) provides superior soft-tissue contrast, which can enhance sensitivity for the detection of mucosal lesions and cystic changes within the maxillary sinus. Nonetheless, differences in specificity between these modalities may affect the accuracy of differentiating maxillary sinus retention cysts (MSRCs) from other pathologies, thereby influencing prevalence estimates derived from MRI-based studies. MSRCs are most often identified as incidental radiological findings, with reported prevalence rates of up to 13% in the adult population on CT and as high as 21% when assessed using MRI [4,28,29]. In the majority of cases, these lesions are asymptomatic, do not result in significant clinical symptoms, and typically do not necessitate surgical intervention. The recommended approach in such instances is conservative management, consisting of regular clinical and radiographic follow-up [29]. To the best of the authors’ knowledge, no studies to date have systematically characterized maxillary sinus retention cysts (MSRCs) using magnetic resonance imaging (MRI), and only a limited number of investigations have explored their features on CT or CBCT imaging.

Analysis of MRI scans from 3092 patients revealed the presence of MSRCs in 176 individuals (5.7%). Previous studies using CT or CBCT imaging have reported MSC prevalence rates ranging from 3.6% to 12.9% measured at the sinus level and from 12.4% to 22% measured at the patient level. The discrepancy between our findings and those of previous studies may be attributed to differences in the clinical indications for CT or CBCT imaging, as well as potential ethnic variations among study populations.

Moreover, in our study, patients aged 31–60 years were more likely to be diagnosed with maxillary sinus retention cysts (MSCRs) compared to other age groups. This finding contrasts with previous studies, which reported the highest prevalence in individuals aged 10–35 years. Nascimento et al. [32] found no statistically significant association between maxillary sinusitis and demographic factors (*p* > 0.05). However, the presence of retention cysts was significantly associated with age. Specifically, younger patients (10–35 years old) were 3.47 times more likely to present with retention cysts compared to older individuals (>51 years old).

Consistent with previous studies involving samples with undermined sinus conditions, we observed the prevalence of mucosal thickening ranging from 29.2% to 56.3%. Our findings fall within this range, with 29.7% of cases showing mild to moderate changes, predominantly mucosal thickening [33,34]. Additionally, in agreement with prior research, MSCRs in our study were more frequently diagnosed in male patients [30]. A potential explanation for this phenomenon may be the greater severity of caries and periapical lesions (PALs) observed in men compared to women, which is consistent with our research [30,33,35].

The results of multicenter epidemiological studies indicate that men generally have poorer oral hygiene habits and a significantly lower awareness of dental prophylaxis. They brush their teeth less often, use dental floss and mouthwash less frequently, and are less likely to attend routine dental appointments [36].

Men are more likely to seek dental care only for acute, severe pain, resulting in a higher prevalence of undiagnosed or untreated pulp necrosis and increased risk of chronic inflammation and odontogenic lesions. In contrast, women generally exhibit greater health awareness, proactive preventive behaviors, and regular dental check-ups [37]. Additionally, estrogen influences bone metabolism, tissue regeneration, and saliva composition, providing protective effects such as acid buffering and antibacterial activity [38]. Men have lower levels of salivary secretory immunoglobulins (mainly IgA), which may increase susceptibility to oral bacterial infections [39]. Thus, both behavioral and biological factors contribute to the higher risk of advanced dental and periodontal pathology observed in men.

In clinical practice, an important aspect of odontogenic pathology is the assessment of maxillary sinus cysts. Due to the anatomical proximity of the roots of molars and premolars to the floor of the maxillary sinus, inflammatory changes in the pulp or periapical tissues can lead to the spread of inflammation into the sinus lumen. In such cases, inflammatory odontogenic cysts may develop, most often as a result of chronic periodontitis. Research data indicate that up to 58% of chronic periapical inflammatory lesions of the teeth may cause secondary involvement of the maxillary sinus [40]. Cysts of dental origin (e.g., radicular cysts) can migrate into the sinus cavity and cause partial or complete filling of the sinus. Studies show that incidental lesions in the maxillary sinuses are detected in most orthodontic patients (60%) in CBCT examinations, which emphasizes the importance of careful analysis of these images. For comparison, Biel et al. [41] found incidental lesions, mainly thickening of the maxillary sinus mucosa (32%), in 82% of patients preparing for implant treatment [42]. Similar results were obtained by Kurtuldu et al. [43] and Gracco et al. [44], who identified abnormalities in the sinuses in approximately 50% of orthodontic patients in CBCT (mucosal thickening: 40.1%, pseudocysts: 10.1%) [42,44].

In cases of coexisting inflammatory changes in the teeth and sinuses, collaboration between ENT and dental teams is recommended. Importantly, untreated dental abnormalities can lead to complications such as chronic maxillary sinusitis, the development of purulent cysts or intrasinus abscesses, and even zygomaticomaxillary rhinosinusitis (ZMR) due to dental sinusitis. Therefore, in the differential diagnosis of any maxillary sinus cyst, especially unilateral in the immediate vicinity of tooth roots, both a dental and radiological evaluation are necessary to determine the possible dental source. Therapeutic management should be individualized; retention cysts without clinical symptoms usually require only observation, while secondary lesions related to dental pathology may be an indication for surgical and dental treatment.

The findings of our study emphasize the importance of radiologists’ descriptions of MRI to its clinical utility. The majority of clinically irrelevant maxillary sinus lesions require to be mentioned in the MRI description; otherwise, patients could lose trust in leading physicians because of no reaction to the radiological findings. Moreover, patients’ curiosity to solve the radiological findings leads to unnecessary further examinations related to a higher radiation dose or expenditures. On the other hand, maxillary cysts related to PALs should be promptly reported as a significant clinical finding that requires rapid dental care to avoid complications such as purulent rhinosinusitis, which could involve meningitis or orbital tissue inflammation. In our study maxillary sinus cysts were frequently identified in the absence of sinonasal or dental symptoms, reinforcing previous findings that lesion size does not reliably predict clinical impact. Several studies have similarly failed to establish a statistically significant correlation between cyst dimensions and symptom severity, suggesting that anatomical location, ostiomeatal involvement, secondary infection, or rate of expansion may play a more decisive role in determining clinical presentation than size alone [45,46].

In the authors’ opinion, future work should focus on prospective studies that stratify cysts not only by size but also by their anatomical relation to adjacent structures such as the ostiomeatal complex, dental roots, or orbital floor in order to clarify predictors of clinical significance. Longitudinal follow-up of incidentally detected, asymptomatic cysts is necessary to determine their natural history and whether certain subgroups are predisposed to progression, symptom development, or the need for surgical intervention.

Although this study provides the first large-scale MRI-based assessment of maxillary sinus lesions, focusing on the prevalence and characteristics of retention cysts and mucosal changes incidentally detected on head MRI scans, it has several limitations. Its retrospective design prevented validation procedures and reproducibility testing for MRI interpretations, including interobserver agreement analysis. Consequently, the consistency and reliability of lesion identification and characterization could not be formally assessed, which may introduce variability in MRI readings and affect the generalizability of the findings. Additionally, although MRI offers excellent soft tissue contrast, its lower specificity compared to CT or CBCT may reduce accuracy in differentiating maxillary sinus retention cysts from other lesions, impacting prevalence estimates and cyst classification. The use of a modified sinus inflammation scale adapted from the Lund–Mackay scale, originally designed for CT, also presents limitations since it has not been fully validated for MRI, which may influence assessment consistency and comparability with other studies. Furthermore, the lack of clinical data correlating imaging findings with patient symptoms or dental status restricts the ability to evaluate the clinical relevance of incidental cysts and their implications for patient management. Future prospective research incorporating standardized MRI reading protocols, inter-rater reliability assessments, and clinical correlations is needed to strengthen the validity and applicability of findings [47,48]. Moreover, chronic rhinosinusitis (CRS) demonstrates important racial and ethnic variations in prevalence, inflammatory patterns, and comorbidities. For example, CRS in Asian populations is more likely to present with neutrophilic or mixed inflammation and less frequently with type 2 (eosinophilic) disease than in Caucasians, which corresponds with lower rates of asthma and aspirin-exacerbated respiratory disease (AERD) comorbidity. In the U.S., CRS is diagnosed less often in Black populations compared with White populations, although allergic fungal rhinosinusitis (AFRS) disproportionately affects Black patients (53.8% of AFRS cases), whereas CRS without nasal polyps is more common in White patients. While race appears to influence the prevalence and subtype distribution of CRS and related sinus conditions, these differences do not impact the clinical significance of the maxillary sinus cyst findings analyzed in this study. Future comparative cohort studies, particularly in Asian and African populations, would be valuable to confirm and extend our observations [46,49,50].

Management pathways for sinus cysts remain a common clinical consideration. Most cysts are asymptomatic and do not require intervention. Surgical removal may be considered in cases where cysts are symptomatic, demonstrate progressive growth, or interfere with planned procedures such as sinus floor elevation for dental implants. Ultimately, management should be individualized, taking into account patient symptoms, imaging findings, and the context of planned surgical interventions [51,52]. Endoscopic endonasal surgery is the preferred approach for symptomatic or large cysts, offering low morbidity and high success rates [53]. In cases where cysts complicate implant procedures, aspiration or digitally guided techniques can safely reduce cyst volume and facilitate implantation [54]. Non-surgical, image-guided aspiration with adjunctive drug injection has also shown promising results in selected cases [55].

It should be emphasized that the vast majority of maxillary sinus retention cysts require no further intervention.

## 5. Conclusions

Changes in maxillary sinuses in asymptomatic patients are quite common because of the vast majority of them. Most changes do not require any intervention. Nevertheless, some maxillary changes of dental origin require a multidisciplinary approach involving both otolaryngologists and dental specialists, though they are infrequent.

Therefore, in any unilateral maxillary sinus lesion with changes in bones, both dental and radiological evaluations are essential. Treatment should be tailored individually; while asymptomatic retention cysts may only require observation, lesions linked to dental pathology often call for combined surgical and dental therapy.

Incidentally detected MRI changes can lead to overtreatment among the non-symptomatic Caucasian population. Awareness of the high incidence of incidental changes among physicians of all specialties may reduce healthcare expenditure and the number of unnecessary medical visits.

## Figures and Tables

**Table 1:** Distribution of maxillary sinus involvement by sex.

	Maxillary Sinus Involvement (T = 1)		
*Sex*	0	1	*p*
Men (*n*)	1031	235	0.001
%	81.4%	18.6%	
Women (*n*)	1568	255	
%	86%	14%	

**Table 2:** Distribution of mucosal thickening limited to the maxillary sinus by age group.

	Mucosal Thickening Affecting only the Maxillary Sinus				
Age Group	18–30 Years	31–60 Years	61–75 Years	76–90 Years	90+ Years
**Patients (*n*)**	58	241	138	53	0
%	11.7%	49.2%	28.2%	10.9%	0.0%

**Table 3:** Classification of sinus changes in Group 1 using the simplified MRI scale.

Changes in Other Paranasal Sinuses	1	2	3	4	5	
**yes**	48	8	2	0	5	***p*** **< 0.001**
	76.19%	12.70%	3.17%	0%	7.94%	
**no**	4	0	0	0	109	
	3.5%	0.00%	0.00%	0%	96.5%	

**Table 4:** Simplified MRI evaluation of sinus changes in Group 1 by sex.

	Simplified MRI Scale					
***Sex***	**1**	**2**	**3**	**4**	**5**	***p***
**Men (*n*)**	30	4	2	0	58	0.502
**%**	31.9%	4.3%	2.1%	0%	61.7%	
**Women (*n*)**	22	4	0	0	55	
**%**	27.2%	4.9%	0.0%	0%	67.9%	

**Table 5:** Classification of non-maxillary paranasal sinus changes in Group 2 using the simplified MRI scale.

	Number	%
0	1995	68.4
1	765	26.2
2	92	3.2
3	62	2.1
5	3	0.1

**Table 6:** Simplified MRI evaluation of sinus changes in Group 2 by sex.

Sex	0	1	2	3	4	5	
**Women (*n*)**	1269	389	55	30	0	1	
**%**	72.85%	22.22%	3.16%	1.72%	0%	0.06%	***p*** **< 0.001**
**Men (*n*)**	725	376	37	32	0	2	
**%**	61.83%	32.11%	3.16%	2.73%	0%	0.17%	

**Table 7:** Distribution of swelling restricted to the maxillary sinus by sex in Group 2.

Sex	Swelling Restricted to the Maxillary Sinus (T = 1)		
	**0**	**1**	***p***
Women (*n*)	1544	199	
%	88.58%	11.42%	**0.003**
Men (*n*)	993	178	
%	84.80%	15.20%	

**Table 8:** Simplified MRI evaluation of sinus changes in Group 2 with swelling limited to the maxillary sinus.

	*n*	%
0	2	0.4%
1	332	67.8%
2	30	6.1%
3	17	3.5%
4	0	0.0%
5	109	22.2%

**Table 9:** Distribution of cysts in group with swelling limited to the maxillary sinus.

	Other Changes	5	*p*
**Men (*n*)**	180	55	0.554
**%**	76.60%	23.40%	
**Women (*n*)**	201	54	
**%**	78.82%	21.18%	

**Table 10:** Age-based distribution of cysts in the group with swelling limited to the maxillary sinus.

	Other	5	*p*
18–30 (*n*)	48	10	
%	82.76%	17.24%	
31–60 (*n*)	176	65	**0.028**
%	73.03%	26.97%	
76–90 (*n*)	109	29	
%	78.99%	21.01%	
90+ (*n*)	48	5	
%	90.57%	9.43%	

**Table 11:** Results of multivariate backward stepwise logistic regression analysis.

Variable	OR (95% CL)	*p*
18–30 years	0.86 (0.54–1.36)	0.516
31–60 years	1	
61–75 years	0.70 (0.49–1.00)	**0.049**
76–90 years	0.33 (0.16–0.65)	**0.001**
M	1.72 (1.27–2.34)	**0.001**

## Data Availability

The data that support the findings of this study are available upon request from the corresponding author.
